# Supplementary material for: Genome Sequencing and Analysis of Nigrospora oryzae, a Rice Leaf Disease Fungus
Source: J Fungi (Basel). 2024 Jan 26;10(2):100. doi: 10.3390/jof10020100 (PMC10890021; doi:10.3390/jof10020100)
Supplement: Supplementary file 1 [file jof-10-00100-s001.zip › Figure and Table.pdf]

Figure and Table

Figure S1. Symptoms on rice of *Nigrospora oryzae* and its morphology on PDA.

Figure S2. Data length distribution diagram of the *Nigrospora oryza* genome. The abscissa represents the length of reads (bp). The vertical coordinate on the left represents the number of reads, corresponding to the green bar chart. The vertical coordinate on the right represents the total number of bases (Mb) contained in reads larger than corresponding length, corresponding to the blue curve; The red dotted line indicates the N50 length of reads.

Figure S3. RNAseq, Denovo and Homolog Wayne fgure. TBtools was used for drawing program (https ://github.com/CJ-Chen/TBtools/ releases)

Figure S4. The classification annotated statistics chart of GO.

Figure S5. The classification annotated statistics chart of KEGG.

Figure S6. The species distribution map of the Nr database was compared to the sequence.

Table S1. Raw data and Clean reads data statistics table.

Table S2. Clean reads length distribution table.

Table S3. BUSCO evaluation statistics.

Table S4. Next generation sequencing data return rate evaluation.

Table S5. Gene prediction results statistics.

Table S6. Statistics of basic gene information.

Table S7. Gene function annotation statistics.

Table S8. The number of co-linear genes in each scaffold of ZQ1.
