# Supplementary figures and images for: Genome Sequencing and Analysis of Nigrospora oryzae, a Rice Leaf Disease Fungus
Source: J Fungi (Basel). 2024 Jan 26;10(2):100. doi: 10.3390/jof10020100 (PMC10890021; doi:10.3390/jof10020100)

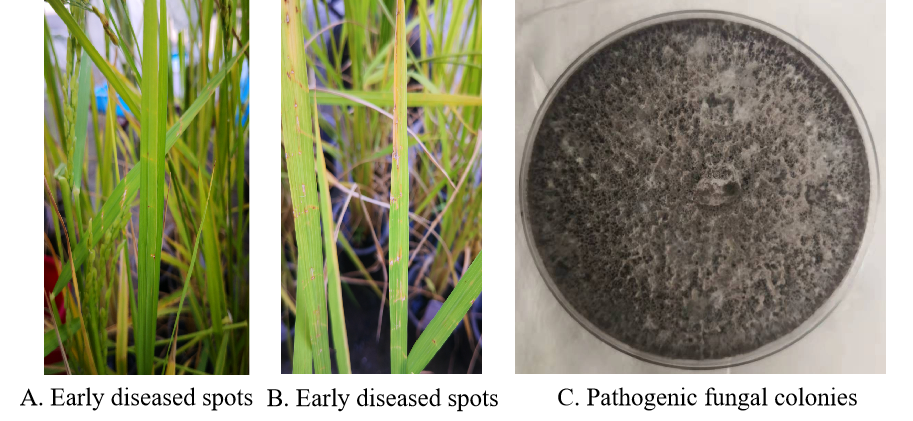

Supplement: Supplementary file 1 [file jof-10-00100-s001.zip › Figure S1.png]

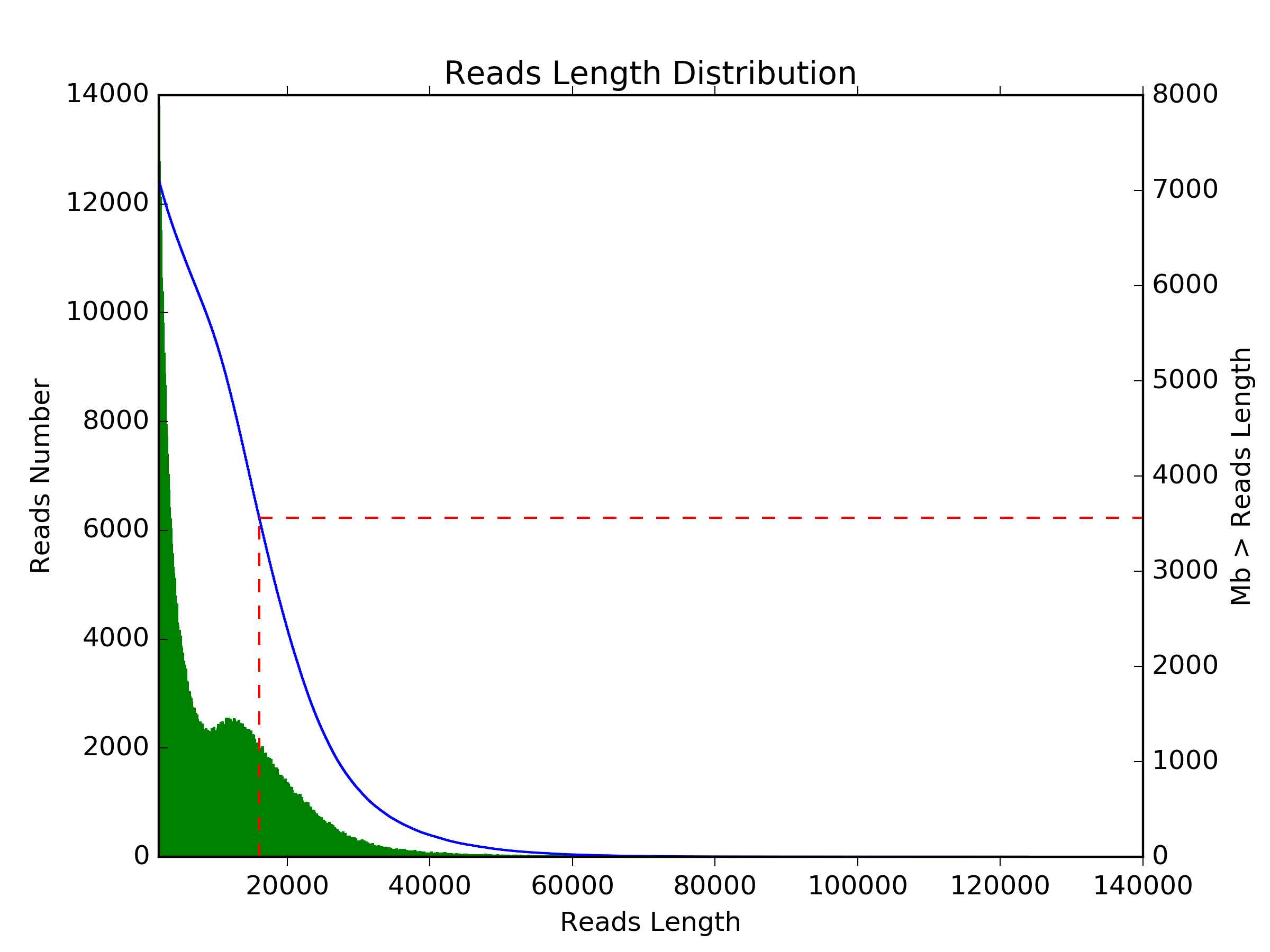

Supplement: Supplementary file 1 [file jof-10-00100-s001.zip › Figure S2.png]

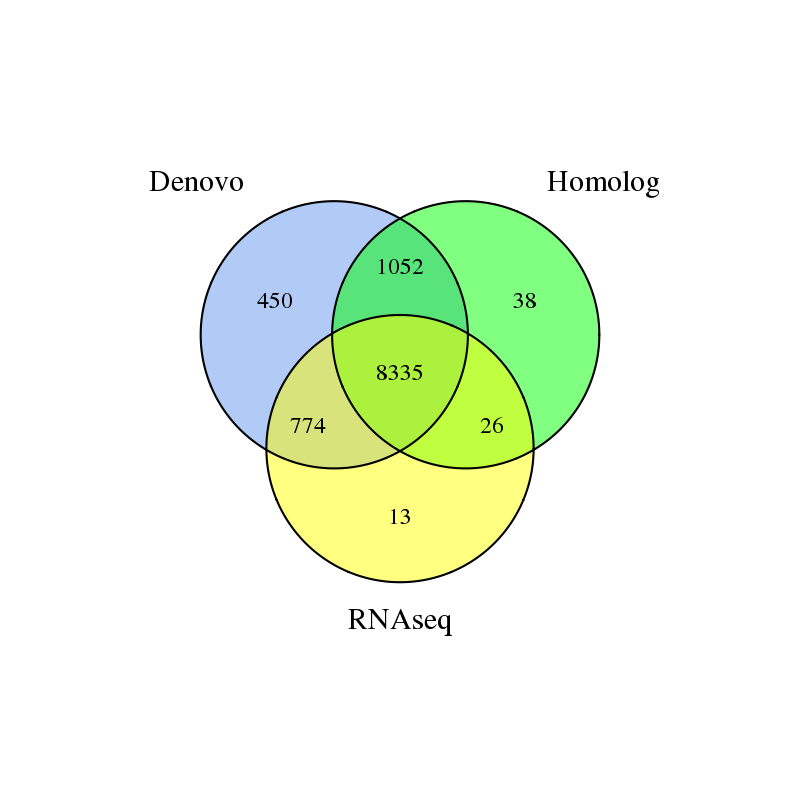

Supplement: Supplementary file 1 [file jof-10-00100-s001.zip › Figure S3.png]

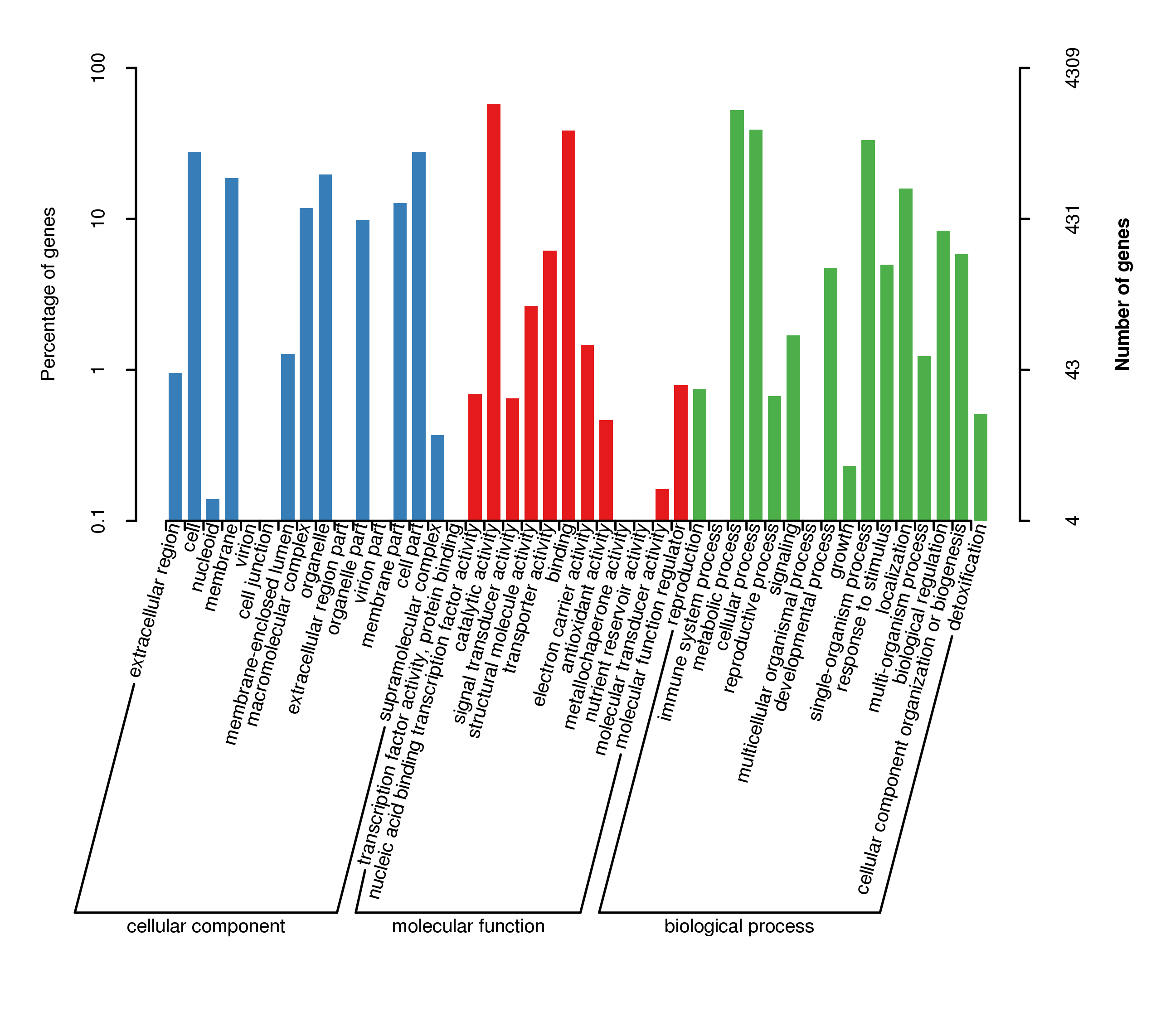

Supplement: Supplementary file 1 [file jof-10-00100-s001.zip › Figure S4.png]

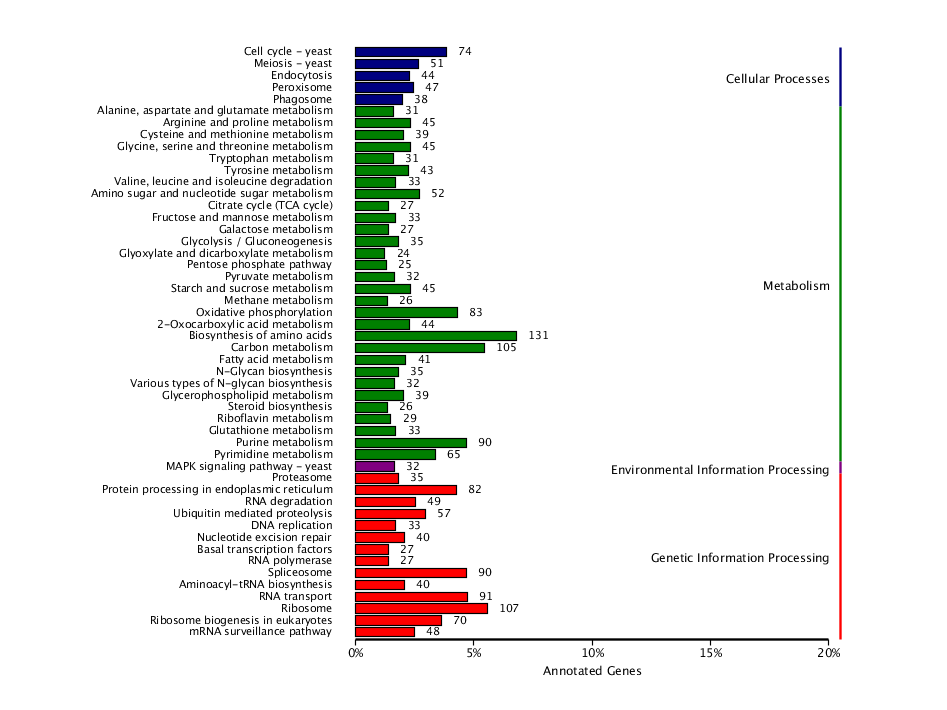

Supplement: Supplementary file 1 [file jof-10-00100-s001.zip › Figure S5.png]

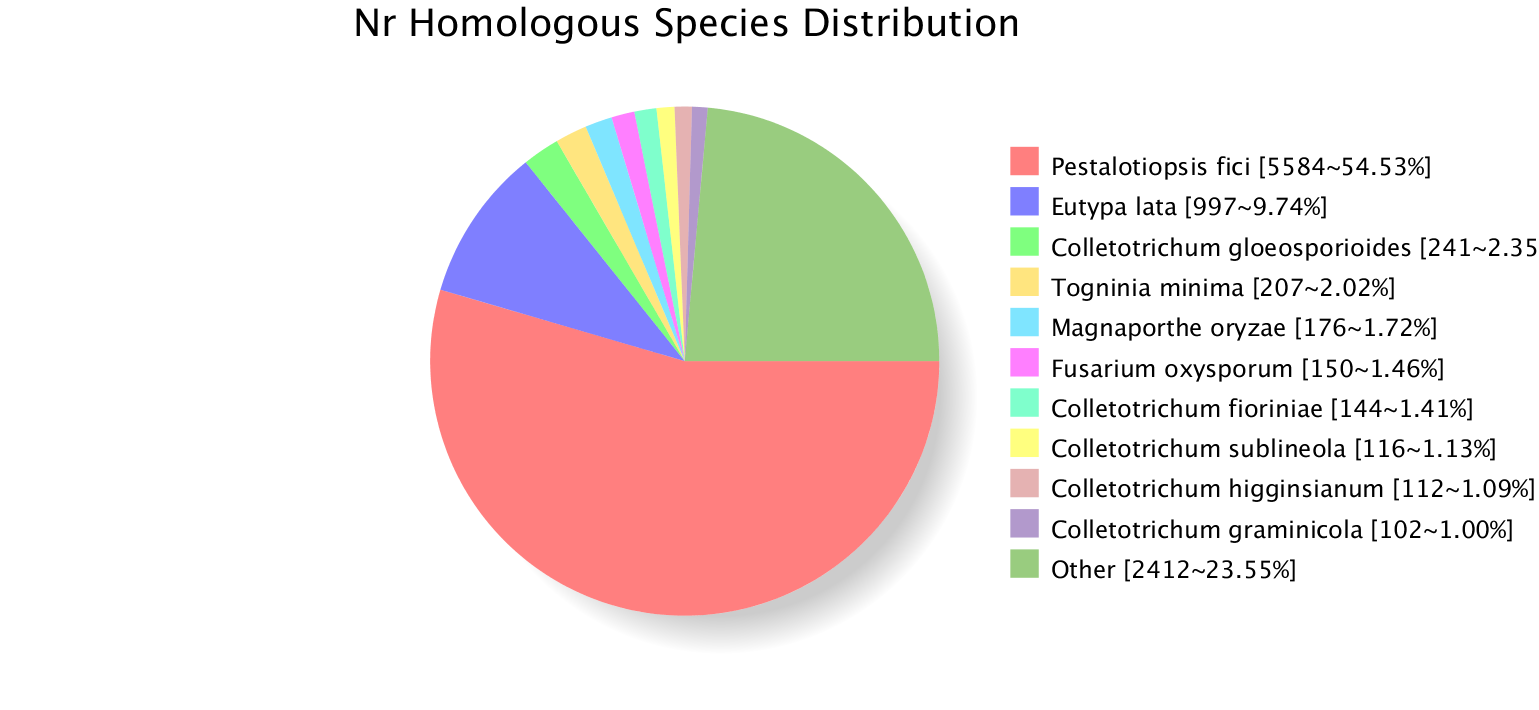

Supplement: Supplementary file 1 [file jof-10-00100-s001.zip › Figure S6.png]
